# IL-1β and TNFα Cooperativity in Regulating IL-6 Expression in Adipocytes Depends on CREB Binding and H3K14 Acetylation

**DOI:** 10.3390/cells10113228

**Published:** 2021-11-19

**Authors:** Areej Al-Roub, Ashraf Al Madhoun, Nadeem Akhter, Reeby Thomas, Lavina Miranda, Texy Jacob, Ebaa Al-Ozairi, Fahd Al-Mulla, Sardar Sindhu, Rasheed Ahmad

**Affiliations:** 1 Immunology & Microbiology Department, Dasman Diabetes Institute, Dasman 15462, Kuwait; Areej.AbuAlRoub@dasmaninstitute.org (A.A.-R.); nadeem.akhter@dasmaninstitute.org (N.A.); reeby.thomas@dasmaninstitute.org (R.T.); texy.jacob@dasmaninstitute.org (T.J.); sardar.sindhu@dasmaninstitute.org (S.S.); 2 Genetics & Bioinformatics, Dasman Diabetes Institute, Dasman 15462, Kuwait; ashraf.madhoun@dasmaninstitute.org (A.A.M.); lavina.miranda@dasmaninstitute.org (L.M.); fahd.almulla@dasmaninstitute.org (F.A.-M.); 3 Animal and Imaging Core Facilities, Dasman Diabetes Institute, Dasman 15462, Kuwait; 4 Medical Division, Dasman Diabetes Institute, Dasman 15462, Kuwait; ebaa.alozairi@dasmaninstitute.org

**Keywords:** interleukin-1β (IL-1β), tumor necrosis factor-alpha (TNFα), adipocytes, interleukin-6 (IL-6), H3K14 acetylation

## Abstract

IL-6 was found to be overexpressed in the adipose tissue of obese individuals, which may cause insulin resistance. However, the regulation of IL-6 in adipocytes in obesity setting remains to be explored. Since IL-1β and TNFα are increased in obese adipose tissue and promote inflammation, we investigated whether cooperation between IL-1β and TNFα influences the production of IL-6. Our data show that IL-1β and TNFα cooperatively enhance IL-6 expression in 3T3L-1 adipocytes. Similar results were seen in human adipocytes isolated from subcutaneous and visceral fat. Although adipocytes isolated from lean and obese adipose tissues showed similar responses for production of IL-6 when incubated with IL-1β/TNFα, secretion of IL-6 was higher in adipocytes from obese tissue. TNFα treatment enhanced CREB binding at CRE locus, which was further enhanced with IL-1β, and was associated with elevated histone acetylation at CRE locus. On the other hand, IL-1β treatments mediated C/EBPβ binding to NF-IL-6 consensus, but not sufficiently to mediate significant histone acetylation. Interestingly, treatment with both stimulatory factors amplifies CREB binding and H3K14 acetylation. Furthermore, histone acetylation inhibition by anacardic acid or curcumin reduces IL-6 production. Notably, inhibition of histone deacetylase (HDAC) activity by trichostatin A (TSA) resulted in the further elevation of IL-6 expression in response to combined treatment of adipocytes with IL-1β and TNFα. In conclusion, our results show that there is an additive interaction between IL-1β and TNFα that depends on CREB binding and H3K14 acetylation, and leads to the elevation of IL-6 expression in adipocytes, providing interesting pathophysiological connection among IL-1β, TNFα, and IL-6 in settings such as obesity.

## 1. Introduction

Obesity is a major health concern that has alarmingly increased worldwide during recent decades [1]. Obesity is characterized by low-grade, chronic inflammation that increases the risk of developing several metabolic disorders such as atherosclerosis, Type 2 diabetes, and hypertension [2,3]. Adipose tissue plays a key role in the development of metabolic inflammation. Inflammation in the adipose tissue is characterized by increased production of proinflammatory cytokines such as IL-1β, IL-6, and TNFα, and an increase in the number of macrophages with a switch in the phenotype from anti-inflammatory M2 to proinflammatory M1 state [4,5,6,7]. These proinflammatory cytokines can impair insulin signaling, and thereby contribute to metabolic dysfunction/insulin resistance [8,9,10,11].

IL-6 has emerged as one of the potential cytokines that link obesity-derived chronic inflammation with insulin resistance. In vitro study shows that IL-6 causes insulin resistance at the cellular level in both primary hepatocytes and HepG2 cells [12]. Increased IL-6 levels have been linked to inhibition of hepatic glycogen synthase, activation of glycogen phosphorylase and lipolysis, and increased triglyceride production [13,14]. Circulating levels of IL-6 have been correlated with adiposity and Type 2 diabetes [15,16,17]. Macrophages and monocytes are considered as a predominant source of IL-6 production.

Recent studies suggest that both the adipose and muscle tissue are important sites of IL-6 production. Adipose tissue has been shown to produce 10–35% of IL-6 in a resting individual, and this production increases with increased adiposity [18], indicating that adipose tissue is a source of the increased circulating IL-6 observed in obesity. IL-6 level is elevated in patients with lipid abnormalities and insulin resistance [19]. Notably, the mechanism(s) triggering abnormally high IL-6 levels in obesity remain unclear. Since elevated levels of IL-1β and TNFα have been previously linked to obesity-induced inflammation and the development of insulin resistance in adipose tissue adipokines [20], we investigated whether these two agents interact to trigger IL-6 production in adipocytes. We found that IL-6 expression was significantly higher in 3T3 L adipocytes or primary human adipocytes treated with IL-1β and TNFα, compared with individual treatment. Furthermore, similar results have been seen in primary adipocytes derived from preadipocytes isolated from lean and obese individuals. Mechanistically, we show that this cooperative and additive effect of IL-1β and TNFα on IL-6 is dependent on CREB binding and H3K14 acetylation.

## 2. Materials and Methods

### 2.1. Differentiation of 3T3-L1 Adipocytes

Mouse 3T3-L1 preadipocytes were purchased from the American Type Culture Collection (Manassas, VA, USA), and seeded onto 6 -well plates (0.25 million cells/well in Dulbecco’s modified Eagle’s medium DMEM-medium (Gibco, Life Technologies, Grand Island, NY, USA) containing 10% FBS (Gibco, Life Technologies, Grand Island, NY, USA), 2 mM glutamine (Gibco, Invitrogen, Grand Island, NY, USA) and 1% penicillin-streptomycin (Gibco, Life Technologies, Grand Island, NY, USA) in a humidified atmosphere containing 5% CO_2_ at 37 °C. Cells were allowed to grow for 2 days, and were then exposed to DMEM containing a differentiation cocktail (5 μg/mL insulin, 0.25 μM dexamethasone, and 0.5 mM IBMX) supplemented with antibiotics and 2 mM L-glutamine in the presence of a vehicle (0.01% DMSO), PGE2 (0.1, 1 and 5 μM) for 2 days. Then, differentiation media were replaced with DMEM containing 10% FBS for 2 days. Finally, the medium was replaced with fresh DMEM, and then adipocytes were stimulated with IL-1β (10 ng/mL; Sigma, Street Saint Louis, MO, USA), TNFα (10 ng/mL; Sigma, Street Saint Louis, MO, USA) or vehicle. After 24 h of treatment, the culture media and adipocytes were harvested. RNA was extracted from the adipocytes and used for the determination of IL-6 mRNA. Culture media were used for IL-6 protein determination.

### 2.2. Differentiation of Human Adipocytes

Human preadipocytes derived from subcutaneous and omental visceral adipose tissues from lean and obese individuals were obtained from ZenBio (Research Triangle Park, NC, USA; catalogue numbers: SP-F-1, OP-F-1, and OP-F-3, respectively). The cells were maintained in preadipocyte growth medium (PM-1, ZenBio, NC, USA). At 80% confluency, cells were differentiated into adipocytes in differentiation medium (DM-2, ZenBio, NC, USA) for 10 days, as described by the manufacturers. Then, the generated primary adipocytes were treated with/without IL-1β, and TNFα alone or in combination.

### 2.3. Nile Red Staining of Lipids

Nile red staining was used to visualize intracellular lipid droplets using fluorescence microscope [21]. Cells were fixed with 4% paraformaldehyde for 15 min and washed three times with 1× PBS. Then, the cells were incubated in 300 nM Nile Red solution for 30 min. Cells were washed three times with 1× PBS. Nuclei were stained with DAPI. Yellow-gold fluorescence was detected using an inverted fluorescence microscope (IX71, Olympus, Japan). The scale bar was 50 µm.

### 2.4. Real-Time RT-PCR

Total cellular RNA was extracted using the RNeasy Mini Kit (Qiagen, Valencia, CA, USA), following the manufacturer’s instructions. Complementary DNA (cDNA) was synthesized using 1 μg of total RNA following the guidelines from the high-capacity cDNA reverse transcription kit (Applied Biosystems, Foster City, CA, USA) [22,23,24,25,26,27]. For each real-time PCR reaction, 50 ng of cDNA template was amplified using Inventoried TaqMan Gene Expression Assay products (mouse IL-6: Hs00446190_m1; 1) Pparg: Mm00440940_m1;Fabp4: Mm00445878_m1; mouse GAPDH:Mm99999915_g; human IL-6: Hs00985639_m1; Ppar g: Hs01115513_m1; PLIN2: Hs00605340_m1; 1; human GAPDH: 4310884E using two gene-specific primers, one TaqMan MGB probe (6-FAM dye-labeled), a TaqMan^®^ Gene Expression Master Mix (Applied Biosystems, Foster City, CA, USA), and a 7500 Fast Real-Time PCR System (Applied Biosystems, Foster City, CA, USA) [28,29,30,31]. The target mRNA levels were normalized against GAPDH mRNA relative to the control, and calculated using the 2^−ΔΔCT^ method [32]. Relative mRNA expression was expressed as fold expression relative to the average of control gene expression. The expression level in the controls was designated as 1 [23,33,34].

### 2.5. ELISA

Secreted IL-6 protein levels were measured in supernatants of TNFα and/or IL-1β stimulated adipocytes using mouse or human IL-6 quantikine ELISA Kits following the manufacturer’s instructions (R&D Systems, Minneapolis, MN, USA).

### 2.6. Confocal Microscopy

For detecting protein expression by confocal microscopy, 3T3 cells were seeded on a coverslip and allowed to settle by incubation for 24 h. Later, cells were treated with 4% paraformaldehyde for 10 min, and permeabilization was performed with 0.25% Triton X-100 in PBS for 10 min. Cells were incubated in blocking reagent Bovine Serum Albumin for 1 h. Anti-IL-6 antibody (GTX17623, Genetex, CA, USA) in 1:200 dilution, anti-tubulin (ab6160, abcam^®^, MA, USA) in 1:200 dilution was used and incubated overnight. Cells were washed three times with PBS–Tris-buffer and incubated in Goat anti-rabbit Alexa Fluor^®^488 (abcam^®^ ab150077, MA, USA) secondary antibody and Goat anti-mouse Alexa Fluor^®^647 (abcam^®^ ab150115, MA, USA) for 1 h. Cells were washed three times with PBS, and then the nucleus was counterstained with 4′,6-diamidino- 2-phenylindole DAPI Vectashield H1500 (Vector Laboratories, CA, USA). Confocal images were collected using an inverted Zeiss LSM710 Spectral confocal microscope (Carl Zeiss, Gottingen, Germany) and a EC Plan-Neofluar 40×/1.30 oil DIC M27 objective lens. After sample excitation using a 405 nm and 488 nm line of an argon ion laser and HeNe 633 laser, optimized emission detection bandwidths were configured using Zeiss Zen 2010 control software. All samples were analyzed using the same parameters, and the resulting color markup of analysis was confirmed for each sample.

### 2.7. Chromatin Immunoprecipitation-qPCR

ChIP assays were performed using a SimpleChIP^®^ Plus Enzymatic Chromatin IP Kit (Cell Signaling Technology Inc., Danvers, MA, USA) [35] with minor modifications. Briefly, 3T3 cells were differentiated into adipocytes, treated with different cytokines, and were crosslinked with 4% formaldehyde (Sigma, Germany). Chromatin was sheared, and a quantity of 50 ug of chromatin was immunoprecipitated with 2 ug antibodies against CREB (Cell Signaling Technology Inc., Danvers, MA, USA), C/EBPβ (Santa Cruz Biotechnology, Dallas, TX, USA), H3K14ac (Cell Signaling Technology Inc., Danvers, MA, USA), or rabbit IgG(Cell Signaling Technology Inc., Danvers, MA, USA), as described in [36]. The immune complexes were captured using magnetic beads (Thermo Fisher Scientific, Waltham, MA, USA). CREB, C/EBPβ, and H3K14ac or IgG-bound chromatins were quantified as a percent chromatin input using QPCR analysis, as described above. To be considered a true association, each ChIP sample was examined for the enrichment of a chromatin locus immunoprecipitated with a specific antibody, and compared with the same chromatin locus immunoprecipitated with a non-specific IgG (ANOVA with *p* < 0.05). Data represent mean ± SD from three independent biological experiments. QPCR reactions were performed using the forward primer 5′-ACTTAAGCACACTTTCCCC-3′, and the reverse primer 5′-ATCTTTGTTGGAGGGTGGG-3′ flanking the CERB and C/EBPβ adjacent bind sites.

### 2.8. Statistical Analysis

Statistical analysis was performed using GraphPad Prism software (La Jolla, CA, USA). Data were shown as mean ± standard error of the mean, unless otherwise indicated. Unpaired Student *t*-tests and one-way ANOVA followed by Tukey’s test were used to compare means between groups. For all analyses, data from a minimum of three sample sets were used for statistical calculation. A *p* value of <0.05 was considered significant. Ns: not significant, * *p* < 0.05, ** *p* < 0.01, *** *p* < 0.001, and **** *p* < 0.0001.

## 3. Results

### 3.1. Stimulation with IL-1β and TNFα Increases IL-6 Expression in Mouse Adipocytes

**Figure 1:**
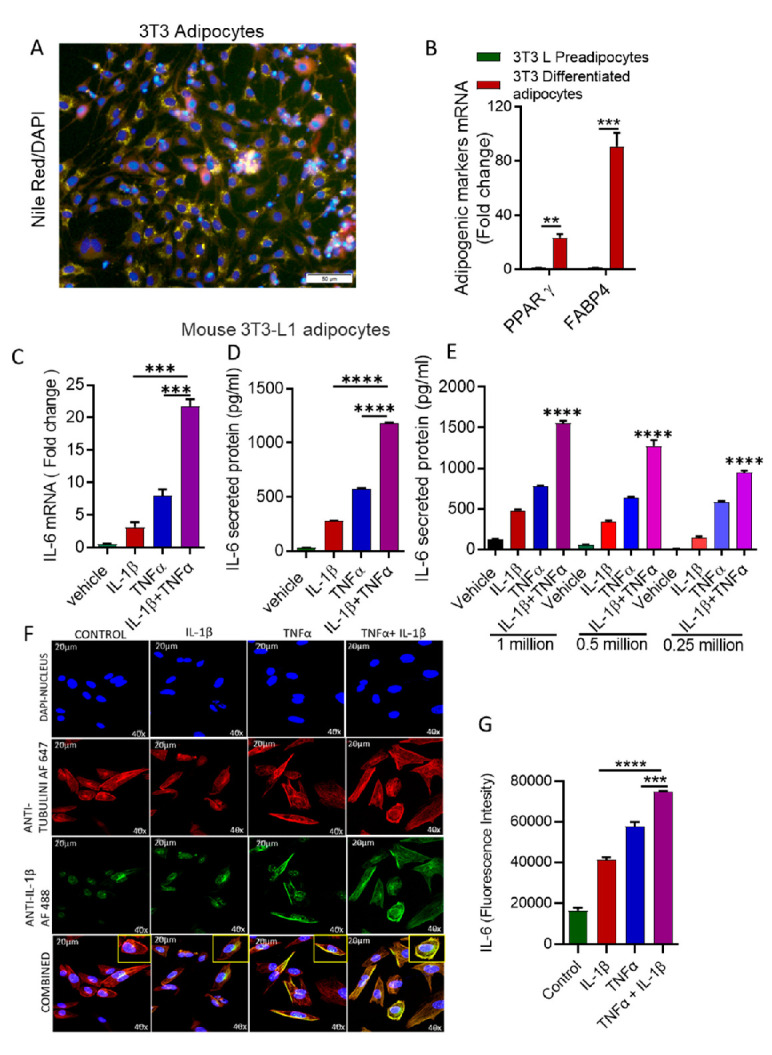
Combined effect of IL-1β and TNFα on IL-6 expression in mouse adipocytes. (**A**,**B**) 3T3 L preadipocytes were differentiated into adipocytes as described in materials & methods. Lipid droplets in adipocytes were determined by using Nile Red staining. Morphology of adipocytes and adipogenic markers were shown. Scale Bar 50 µm. Mouse 3T3-L adipocytes were stimulated with IL-1β (10 ng/mL) and TNFα (10 ng/mL) alone or in combination for 24 h. Cells and culture media were collected. (**C**) Total RNA was extracted from the cells and IL-6 mRNA was quantified by real time PCR. Relative mRNA expression was expressed as a fold change. (**D**) Secreted IL-6 protein in culture media was determined by ELISA. (**E**) Different number of cells (1, 0.5, 0.25 million) were treated with IL-1β (10 ng/mL) and TNFα (10 ng/mL) alone or in combination for 24 h. Cells and culture media were collected, Secreted IL-6 protein in culture media was determined by ELISA. (**F**) 3T3 adipocyte cells were stained for confocal microscopy, as described in the Materials and Methods section. IL-6 expression is shown by green fluorescence (inset), whereas nuclei are stained blue with DAPI (original magnification ×40). Scale Bar 20 µm. (**G**) IL-6 fluorescence intensity was determined for 10 random images. The results obtained from three independent experiments are shown. All data are expressed as mean ± SEM (*n* = 3). ** *p* < 0.01, *** *p* < 0.001, **** *p* < 0.0001.

IL-1β and TNFα levels were elevated, along with high levels of IL-6 in obese adipose tissue [15,37,38]. To assess whether IL-1β and TNFα together induced IL-6 production in adipocytes, we used differentiated mouse 3T3L-1 preadipocytes into adipocytes. Differentiation of the preadipocytes into adipocytes was confirmed by Nile Red staining of lipids (Figure 1A) and expression of markers for adipogenesis (PPARγ, FABP4: Figure 1B). 3T3 adipocytes were challenged either by IL-1β and TNFα alone, or in combination, and IL-6 mRNA and protein were determined. The co-stimulation with IL-1β and TNFα resulted in substantially greater IL-6 expression at both mRNA and protein levels (Figure 1C–E). The effect of the combination of IL-1β and TNFα on IL-6 production was greater than the sum of the individual effects of IL-1β and TNFα, demonstrating additive effects. This elevated IL-6 expression was also determined by confocal microscopy (green fluorescence) (Figure 1F,G).

### 3.2. Stimulation with IL-1β and TNFα Increases IL-6 Expression in Human Primary Adipocytes

**Figure 2:**
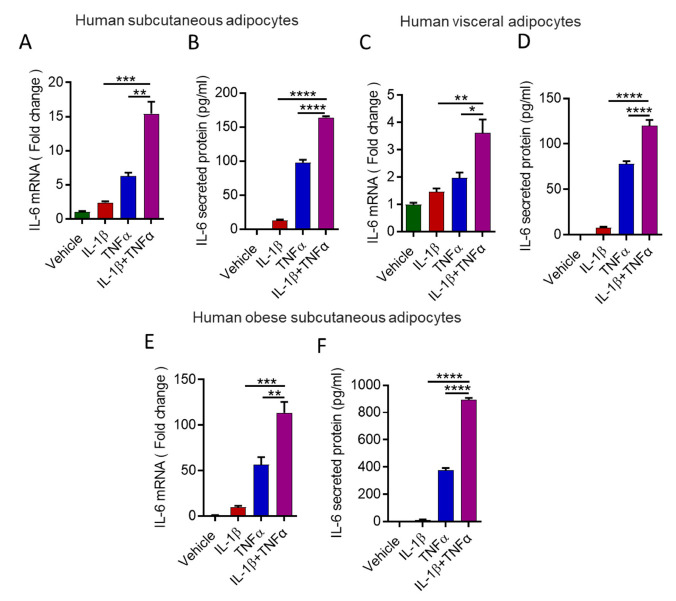
Combined effect of IL-1β and TNFα on IL-6 expression in human adipocytes. Human primary subcutaneous adipocytes were stimulated with IL-1β (250 pg/mL) and TNFα (250 pg/mL) alone or in combination. Cells and culture media were collected. (**A**) Total RNA was extracted from the cells and IL-6 mRNA was quantified by real time PCR. Relative mRNA expression was expressed as a fold change. (**B**) Secreted IL-6 protein in culture media was determined by ELISA. (**C**,**D**). Human primary visceral adipocytes were stimulated with IL-1β and TNFα alone or in combination. Cells and culture media were collected, and IL-6 were determined. (**E**,**F**) Human primary adipocytes isolated from obese adipose tissue treated as described earlier. Cells and culture media were collected, and IL-6 was determined. Data are expressed as mean ± SEM (*n* = 3). * *p* < 0.05, ** *p* < 0.01, *** *p* < 0.001, **** *p* < 0.0001.

Next, we assessed whether a similar cooperative relationship was observed between IL-1β and TNFα in primary human adipocytes. To this end, preadipocytes of lean individuals were differentiated into adipocytes. Differentiation of the preadipocytes into adipocytes was confirmed by Nile red staining and expression of markers for adipogenesis (Supplementary Figure S1A,B). Primary human adipocytes of lean individuals were incubated with IL-1β /TNFα, and IL-6 gene expression was determined. Similar to mouse adipocytes, human differentiated adipocytes derived from either preadipocytes isolated from subcutaneous or visceral adipose tissues showed a cooperative effect of IL-1β and TNFα on IL-6 expression at gene and protein levels (Figure 2A–D). To expand on these findings, we incubated adipocytes (Supplementary Figure S2A,B) isolated from obese adipose tissue with IL-1β and TNFα. However, despite similar cooperativity have been seen for IL-6 production in response to IL-1β and TNFα, the production of IL-6 was noted relatively high (Figure 2E,F).

### 3.3. IL-1β/TNFα Stimulation Increases CREB Binding at IL-6 Promoter

**Figure 3:**
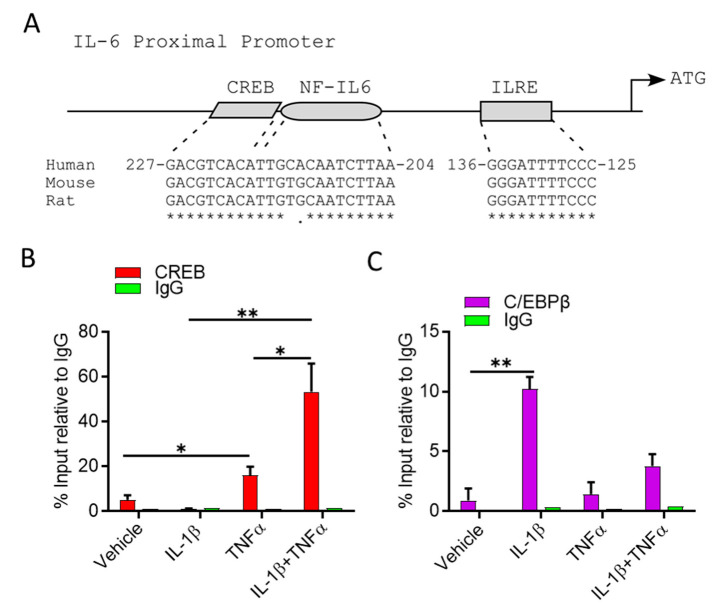
Combined treatment of IL-1β and TNFα increases CREB binding at IL-6 promoter. (**A**) IL-6 promoter contains an adjacent CREB and C/EBPβ binding sites. Chromatin from adipocytes treated with IL-1β, TNFα alone or in combination was subjected to ChIP with antibodies against (**B**) CREB or (**C**) C/EBPβ followed by qRT-PCR. CREB or C/EBPβ occupancy at IL-6 promoter was determined. Data are expressed as mean ± SEM (*n* = 3). * *p* < 0.05, ** *p* < 0.01.

IL-1β and TNFα are cytokines that exert their biological function via downstream signalling pathways, activating transcription factors that in turn regulate gene expression. Studies have been shown that TNFα increases the DNA binding capacity of cyclic AMP Response Element-binding protein (CREB) to CRE-like element (CRE) motif [39], whereas IL-1β enhancing CCAAT/enhancer binding protein beta (C/EBPβ) binds to a consensus site named nuclear factor that specifically binds to an IL1-responsive element in the IL-6 gene (NF-IL6) [40]. Notably, adjacent CRE and NF-IL6 motives are mapped at the IL6 proximal promoter at nucleotides 204–227 upstream from the translation start site (Figure 3A) [41].

Since IL-1β and TNFα cooperatively induced IL-6 transcripts, we examined the ability of CREB and C/EBPβ to bind to the endogenous IL-6 promoter in adipocytes treated with TNFα, IL-1β, alone or in combination, using chromatin immunoprecipitation (ChIP), followed by Q–PCR. Relative to the vehicle control treatment, CREB and C/EBPβ bindings to their corresponding motives were significantly enhanced by 5- and 10-fold in response to TNFα and IL-1β treatments, respectively (Figure 3B,C). Interestingly, treatment with both stimulatory factors significantly augmented CREB bindings 60-fold, relative to vehicle control, but not C/EBPβ bindings (Figure 3B,C). Together, these data suggest that IL-1β generates temporal binding of C/EBPβ to the NF-IL-6 consensus, which facilitates CREB binding in response to TNFα treatment. Furthermore, ERK1/2 are involved as the upstream regulators of CREB and C/EBPβ signalling, following cooperative stimulation of mouse adipocytes by IL-1β and TNFα. It is further shown that ERK1/2 inhibitors (PD98059 and U0126) block the cooperative induction of IL-6 gene end secreted protein expression (Supplementary Figure S3A,B).

### 3.4. Cooperative Induction of IL-6 in Adipocyte Requires H3K14 Acetylation

**Figure 4:**
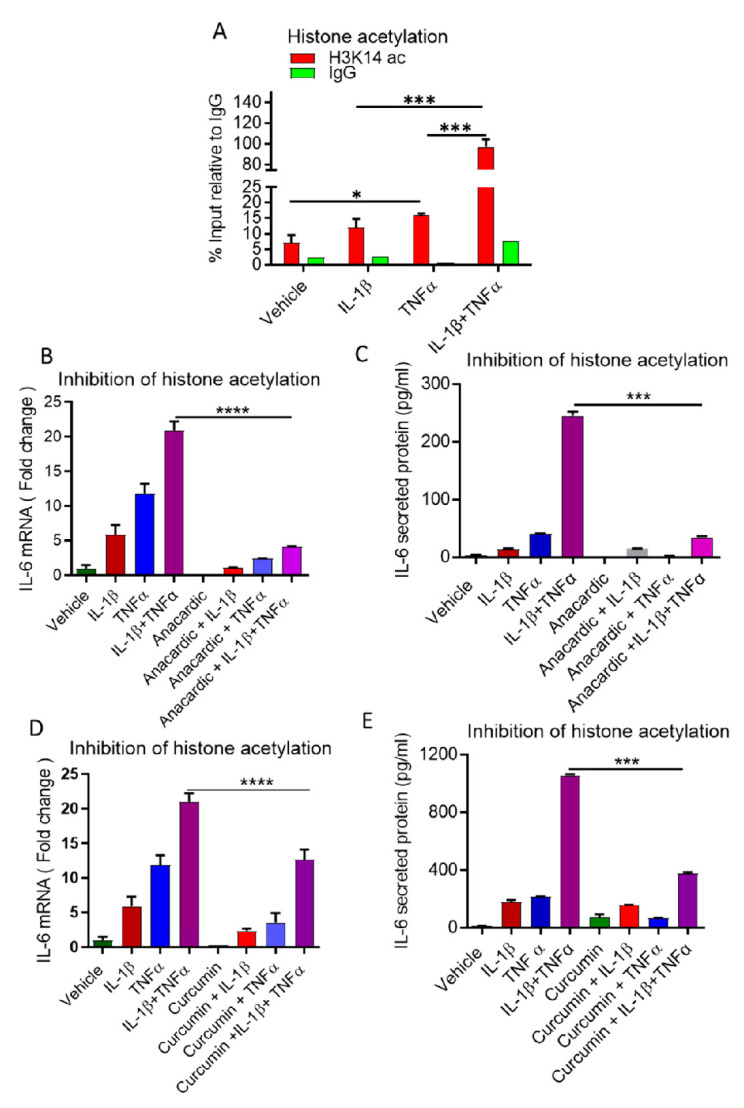
Combined treatment of IL-1β and TNFα increased H3K14 acetylation. (**A**) 3T3-L adipocytes were incubated with vehicle, IL-1β and TNFα, alone or in combination, for 5 h. Histone acetylation at IL-6 promoter was determined by analyzing chromatin that was immunoprecipitated with anti-acetylated histone H3 lysine14 (H3K14ac) or IgG (as a control) antibody. Levels of histone modifications were measured using PCR primers for IL-6 proximal promoter (**B**,**C**) 3T3-L1 adipocytes were incubated with anacardic acid (HATs inhibitor; 4 μM) for 1 h, followed by the stimulation with IL-1β, TNFα or IL-1β/TNFα for 24 h. IL-6 mRNA and secreted protein were determined by qRT-PCR and ELISA, respectively. (**D**,**E**) 3T3-L1 adipocytes were incubated with curcumin (HATs inhibitor; 20 μM) for 2 h, followed by stimulation with IL-1β, TNFα or IL-1β/TNFα for 24 h. IL-6 mRNA and secreted protein were determined by qRT-PCR and ELISA, respectively. Data are expressed as mean ± SEM (*n* = 3). * *p* < 0.05, *** *p* < 0.001, **** *p* < 0.0001.

In response to stimuli, histone acetylation mediates epigenetic modification at IL-6 promoter and induces transcription [42]. To determine if histone acetylation levels were changed at IL-6 proximal promoter in response to IL-1β and TNFα, alone or in combination, at the same locus flanking CRE and NF-IL6 motives, ChIP was performed with antibodies against acetylated H3K14ac as indicative of actively transcribed chromatin [36,43]. Interestingly, the level of H3K14ac was significantly higher at the proximal IL-6 promoter when treated with both IL-1β and TNFα, as compared to individual treatment (Figure 4A). These results indicate that IL-6 expression is mainly dependent on the binding of both CREB and C/EBPβ to their corresponding binding sites in response to IL-1β and TNFα treatments.

To confirm the role of histone acetylation, we examined whether the inhibition of histone acetyl transferases (HATs) influences IL-lβ/ TNFα additive effect on IL-6 secretion. Prior to the treatment of the cytokines, adipocytes were pre-treated with the pharmacological HAT inhibitor anacardic acid or the naturally occurring inhibitor curcumin, both of which have been shown to inhibit HATs in vitro [44,45]. Notably, both inhibitors significantly reduced IL-6 mRNA and IL-6 secretion from cells treated with either TNFα alone or in combination with IL-1β (Figure 4B–E). No alteration in IL-6 secretion was observed in cells incubated with the inhibitors prior IL-1β stimulation (Figure 4B–E), indicating a secondary role for IL-1β in the process of IL-6 induction and secretion.

**Figure 5:**
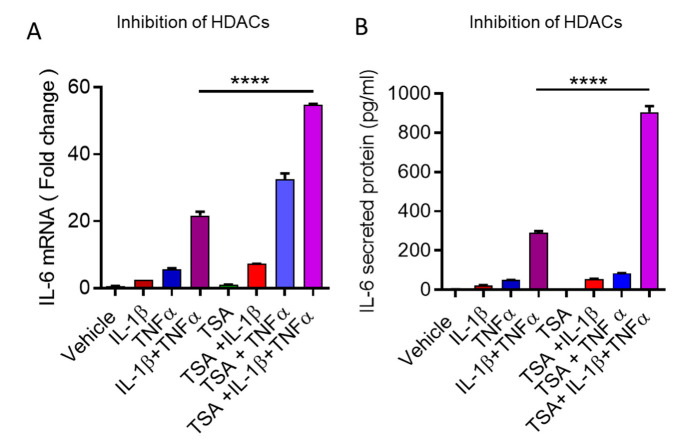
Trichostatin A (TSA) further enhances IL-1β/TNFα expression of IL-6. Cells were treated with TSA (10 nM) for 4 h before stimulation with vehicle, IL-1β, TNFα, or IL-1β + TNFα for 24 h. (**A**,**B**) IL-6 mRNA and secreted protein were determined by qRT-PCR and ELISA, respectively. Data were expressed as mean ± SEM. **** *p* < 0.0001.

Trichostatin A (TSA) is an HDAC inhibitor, and plays a significant role in increasing histone acetylation and gene transcription [46,47]. To determine whether TSA can promote IL-6 transcription and secretion, adipocytes were treated with TSA prior to the treatment of IL-1β/TNFα. Our results show that IL-1β/TNFα additive effect on IL-6 expression, and secretion was further increased in a significant manner (Figure 5A,B).

## 4. Discussion

IL-6 is known as one of the critical cytokine among other immune-modulating cytokines that are dysregulated most frequently in obesity, and increased circulatory levels of IL-6 have been consistently documented in obese mice and humans [16]. IL-6 plays a role in T-cell activation, tissue infiltration, and maintenance of memory responses, as well as orchestrates cellular insulin resistance [48]. Circulating IL-6 levels were found to be related to body mass indices and lipid profiles in overweight and obese individuals [49].

Similarly, IL-1β and TNFα are two other well-known adipokines that are found to be upregulated in the circulation as well as in adipose tissue in obesity, and are known to play key roles in metabolic inflammation and development of insulin resistance, while the inhibition of IL-1β and/or TNFα led to an amelioration in insulin resistance [50,51,52]. Not surprisingly, substantial evidence supports both the higher circulatory levels and adipose expression of these proinflammatory cytokines (IL-6, TNFα and IL-1β) in obesity settings [15,38,53].

Notably, IL-6 regulation in adipocytes in obesity setting remains unclear. Since TNFα and IL-1β expression is elevated in obese adipose tissue, which plays a pivotal role in the maintenance of chronic low-grade inflammation, we determined whether the IL-1 β/TNFα cooperativity could amplify IL-6 expression in mouse and human adipocytes. We show, for the first time to our knowledge, that IL-1β and TNFα co-induce increased IL-6 expression in 3T3L-1 mouse adipocytes, as well as in the human adipocytes from subcutaneous and visceral fat origin. Previously, IL-1β-mediated induction of IL-6 has been shown in numerous cell types, including MCF7 human breast carcinoma cells [54], human mast cells [55], fibroblasts, endothelial cells, keratinocytes, and peripheral blood monocytes [56]. Likewise, TNFα also induces IL-6 production in a variety of cells, such as glioma cells, osteoblasts, and vascular smooth muscle cells, through distinct transduction pathways [57,58,59,60]. However, our findings that IL-1β and TNFα cooperatively amplify the expression of IL-6 in human and mouse adipocytes are novel, and not only show that both IL-1β and TNFα could induce IL-6 expression in adipocytes, but also demonstrate a mechanism as to how IL-1β/TNFα co-expression could lead to elevations in IL-6 levels in obesity setting.

It is of further interest to note that, although the pattern of IL-6 production was similar in adipocytes derived from lean and obese adipose tissues, the IL-6 production co-induced by IL-1β and TNFα was much higher in adipocytes from obese tissue, which implies that obesity-associated changes may reprogram adipocytes for increased IL-6 production following exposure to these two prototypical inflammatory cytokines. The clinical data implicating expression of cytokines/chemokines in obesity/T2D from our group and others concur with this argument [61].

IL-1β or TNFα activates downstream ERK1/2 and C/EBPβ in various cells [62,63,64], and our data show that ERK1/2 are involved as the upstream regulators of CREB and C/EBPβ signaling following cooperative stimulation of mouse adipocytes by IL-1β and TNFα. It is further shown that ERK1/2 inhibition block the cooperative induction of IL-6 gene end secreted protein expression. Regarding further molecular mechanisms underlying IL-1β/TNFα induced IL-6 gene expression, previous studies of IL-6 proximal promoter highlighted the importance of the first 300 base pairs nucleotides upstream of the translation start site. This locus contains consensus sites for CERB, NF-kB, C/EBPβ, and AP-1 transcription factors are often required for IL-6 expression, depending on the cell type and stimuli [65,66,67]. The Interleukin Response Element (ILRE), a short 11 base-pair sequence, located 125 nucleotides upstream from the transcription start site of IL-6 (Figure 3A), was found to be initial for transactions activation. Promoter mapping studies have indicated that ILRE is crucial for TNFα and IL-β response [68] and co-transfection of human monocytic cell line U937 with C/EBPβ and the NF-kB p65 subunit resulted in strong synergistic activation of an IL-6 promoter-reporter constructs [69]. Interestingly, promoter deletion mutants at ILRE site resulted in IL-6- transcription abolishment and a loss of induction by either C/EBPβ or the NF-kB [68,69].

In this study, we used specific primers directed toward a specific locus within IL-6 proximal promoter, which contains adjacent CRE and NF-IL6 motifs and is located 200 bp upstream from the IL-6 translation initiation site [41,70]. Site directed mutations within CRE or NF-IL6 motifs reduced IL-6 promoter activity in luciferase assays, and eradicated CREB and C/EBPβ bindings in electrophoretic mobility shift assays [65,71], suggesting that these motifs are crucial for IL-6 transcription regulations. Therefore, we investigated the importance of this regulatory region IL-6 gene expression in response to TNFα and IL-1β signaling pathways. The synergetic action of TNFα and IL-1β was further defined using ChIP-qPCR analysis, and showed that the endogenous CREB and C/EBPβ transcription factors were differentially bound to their consensus DNA binding sites at the IL-6 proximal promoter. Since remodeling of chromatin within the nucleus is controlled by the degree of acetylation/deacetylation of histone residues on the histone core around which DNA is coiled [72], we observed that CREB binding was associated with elevated levels of histone 3 acetylation, suggesting active transcription, at least in part that the described locus of IL-6 proximal promoter. Furthermore, we found that inhibition of acetyltransferases (HATs) by anacardic acid and curcumin [73], which promote acetylation, resulted in suppression of the additive effect of IL-1β and TNFα on IL-6 production. However, inhibition of HDACs further enhanced the synergistic expression and production of IL-6 in response to IL-1β/TNFα. These findings are clearly highlighting the importance of the acetylation in this cooperativity. Another study by Yan et al. showed that HDAC9 deficiency led to reduced inflammation. It could be possibly a cell-type dependent mechanism that differentially regulates an epigenetic switch in adipocytes vs. effector T lymphocytes [74]. Interestingly, the upregulation of IL-6 gene expression in response to TNFα and IL-1β treatments indicated that a direct interaction of their downstream effectors CREB and C/EBPβ with IL-6 regulatory region and the specificized locus.

**Figure 6:**
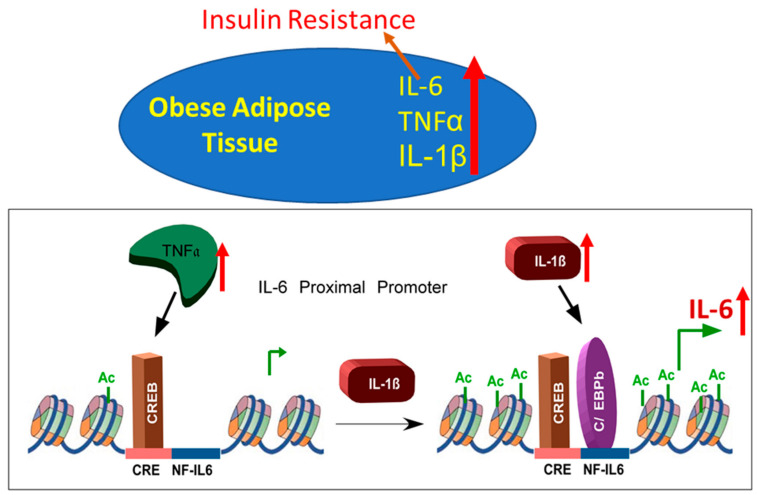
Schematic illustration of signaling pathway underlying IL-1β/TNFα-induced expression of IL-6 in adipocytes.

Notably, treatment with both cytokines induced CREB binding to CRE remarkably, but not C/EBPβ binding to the NF-IL6 motif. Together, these data suggest that, although both TNFα and IL-1β are sufficient to induce IL-6 promoter activity, both signaling pathways are required for IL-6 active transcription. In the context of our data, we propose that IL-1β may generate a temporal binding of C/EBPβ to NF-IL-6 consensus, which facilitates CREB binding in response to TNFα treatment (Figure 6). Meanwhile, TNFα and IL-1β treatments alone are not sufficient to recruit the binding of their alternate transcription factors, at least in part at this regulatory region.

## 5. Conclusions

Our results show that there is a cooperative interaction between IL-1β and TNFα that requires CREB binding and H3K14 acetylation, and leads to the activation of IL-6 expression in adipocytes, providing interesting pathophysiological network among IL-1β, TNFα, and IL-6 in metabolic inflammatory settings such as obesity.

## Data Availability

The data presented in this study are available on request from the corresponding author.

## Supplementary Materials

The following are available online at https://www.mdpi.com/article/10.3390/cells10113228/s1. Figure S1: Characterization of differentiated human preadipocytes isolated from lean adipose tissue. Figure S2: Characterization of differentiated human preadipocytes isolated from obese adipose tissue. Figure S3A: IL-1β/TNFαcooperatively enhances ERK1/2 phosphorylation. Figure S3B,C: Inhibition of ERK1/2 blocks the cooperative effect of IL-1β/TNFα on IL-6 expression and protein secretion.
