# Supplementary material for: IL-1β and TNFα Cooperativity in Regulating IL-6 Expression in Adipocytes Depends on CREB Binding and H3K14 Acetylation
Source: Cells. 2021 Nov 19;10(11):3228. doi: 10.3390/cells10113228 (PMC8619559; doi:10.3390/cells10113228)
Supplement: Supplementary file 1 [file cells-10-03228-s001.zip › cells-1465578-supplementary.pdf]

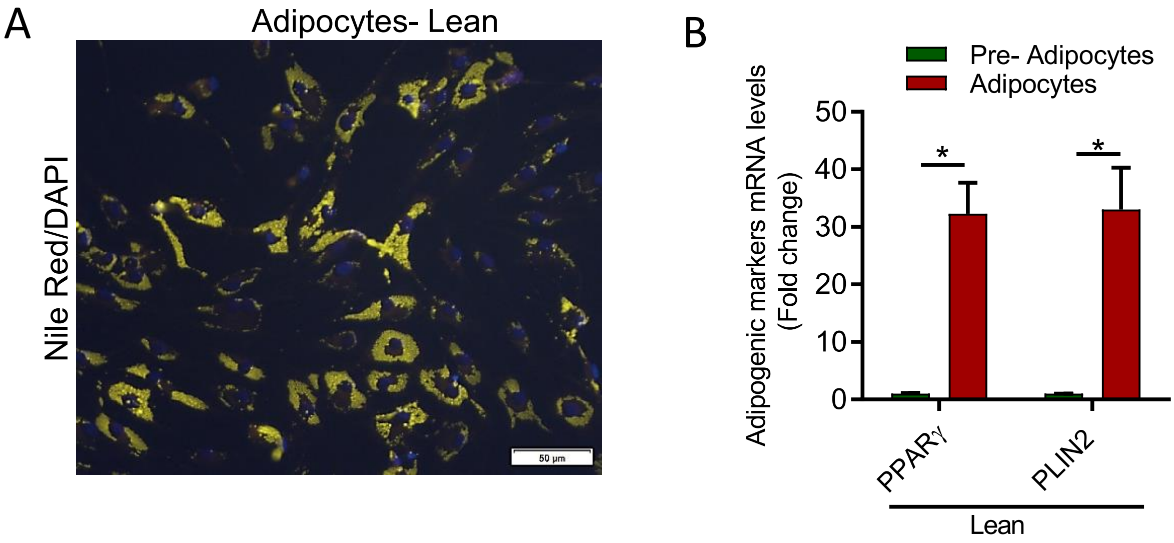

Figure S1. (A, B) Characterization of differentiated human preadipocytes isolated from lean adipose tissue. Human preadipocytes were differentiated into adipocytes as described in materials & methods. Lipid droplets in adipocytes were determined by using Nile Red staining and adipogenic markers were determined by qRT-PCR. Morphology of adipocytes, scale bar 50μm, and adipogenic markers were shown. Data are expressed as mean ± SEM ($n = 3$). * $p < 0.05$.

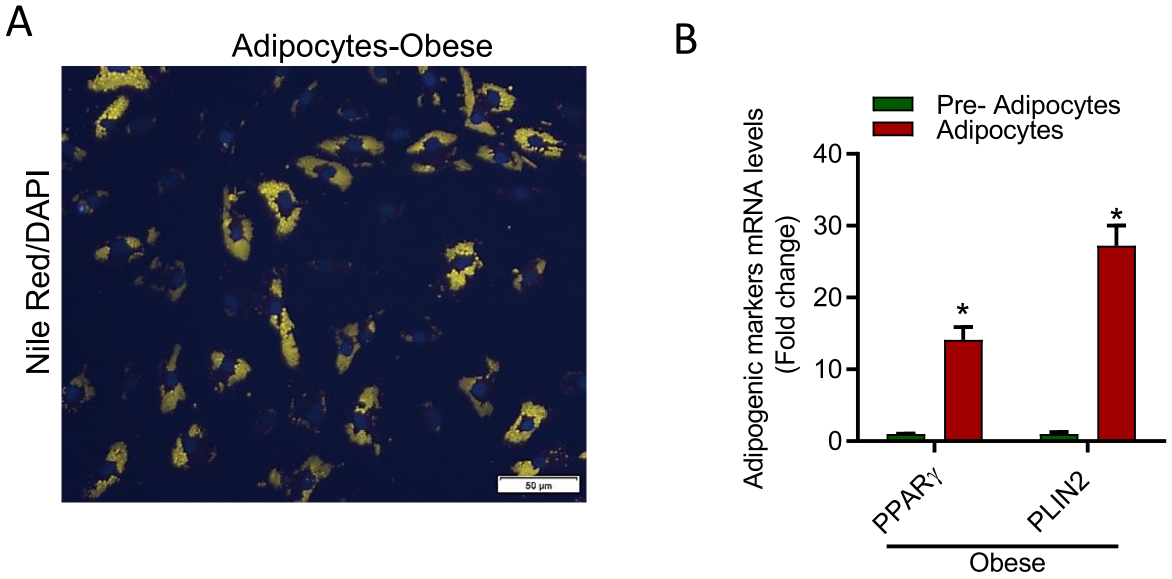

Figure S2. (A, B) Characterization of differentiated human preadipocytes isolated from obese adipose tissue. Human preadipocytes were differentiated into adipocytes as described in materials & methods. Lipid droplets in adipocytes were determined by using Nile Red staining and adipogenic markers were determined qRT-PCR. Morphology of adipocytes, 50μm, and adipogenic markers were shown. Data are expressed as mean ± SEM (n = 3). * $p < 0.05$.

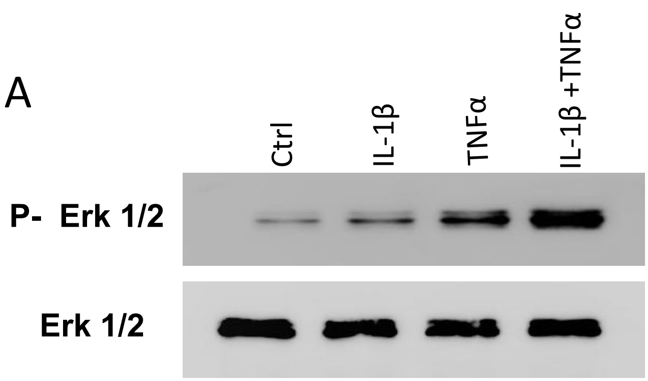

Figure S3A. IL-1β/TNFα cooperatively enhances ERK1/2 phosphorylation.
3T3 adipocytes were stimulated with Vehicle, IL-β1, TNFα alone or in combination.
Following different treatments, 3T3 adipocytes were harvested and incubated for 30 min with lysis buffer (10× Lysis Buffer, Cell Signaling Technology Inc., Danvers, MA, USA). The protein lysates were prepared and resolved using 12% SDS-PAGE. Cellular proteins were transferred to Immuno-Blot PVDF membranes (Bio-Rad Laboratories, Hercules, CA, USA) by electro blotting. The membranes were then blocked with 5% non-fat milk in PBS for 1 h followed by incubation with primary antibodies against p-ERK1/2 and ERK1/2 and JNK at a 1:1000 dilution at 4 °C overnight. All the primary antibodies were purchased from Cell Signaling (Cell Signaling Technology Inc., Danvers, MA, USA). The blots were then washed three times with TBS-T and incubated for 2 h with HRP-conjugated secondary antibody (Promega, Madison, WI, USA). Immunoreactive bands were developed using an Amersham ECL Plus Western Blotting Detection System (GE Health Care, Buckinghamshire, UK) and visualized with a Molecular Imager® (VersaDocTM MP Imaging Systems, Bio-Rad Laboratories, Hercules, CA, USA)

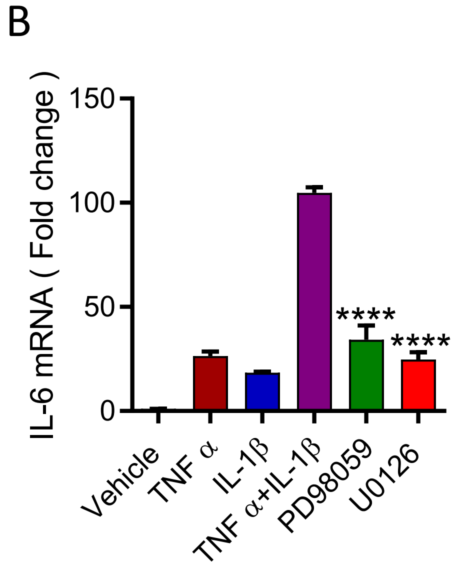

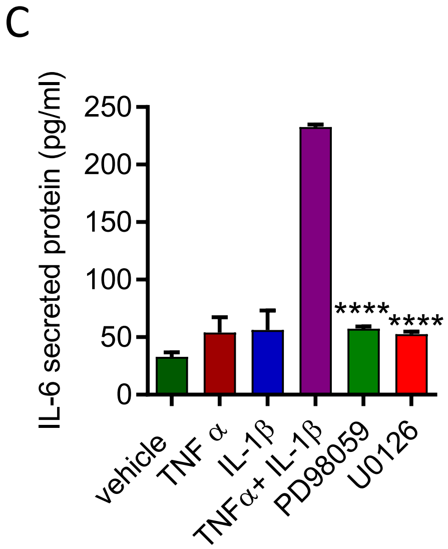

Figure S3B,C. Inhibition of ERK1/2 blocks the cooperative effect of IL-1β/TNFα on IL-6 expression and protein secretion. 3T3-L1 adipocytes were incubated with PD98059 (10 μM) or U0126 (10μM) for 1h followed by the stimulation with IL-1β, TNFα or IL-1β/TNFα for 24 h. IL-6 mRNA and secreted protein were determined by qRT-PCR and ELISA, respectively. Data are expressed as mean ± SEM (n = 3). ****p < 0.0001.
